# Bacterial release from pipe biofilm in a full-scale drinking water distribution system

**DOI:** 10.1038/s41522-019-0082-9

**Published:** 2019-02-22

**Authors:** Sandy Chan, Kristjan Pullerits, Alexander Keucken, Kenneth M. Persson, Catherine J. Paul, Peter Rådström

**Affiliations:** 1 Applied Microbiology, Department of Chemistry, Lund University, P.O. Box 124, SE-221 00 Lund, Sweden; 2 Sweden Water Research AB, Ideon Science Park, Scheelevägen 15, SE-223 70 Lund, Sweden; 3 Sydvatten AB, Hyllie Stationstorg 21, SE-215 32 Malmö, Sweden; 4 Water Resources Engineering, Department of Building and Environmental Technology, Lund University, P.O. Box 118, SE-221 00 Lund, Sweden; 5 Vatten & Miljö i Väst AB, P.O. Box 110, SE-311 22 Falkenberg, Sweden

## Abstract

Safe drinking water is delivered to the consumer through kilometres of pipes. These pipes are lined with biofilm, which is thought to affect water quality by releasing bacteria into the drinking water. This study describes the number of cells released from this biofilm, their cellular characteristics, and their identity as they shaped a drinking water microbiome. Installation of ultrafiltration (UF) at full scale in Varberg, Sweden reduced the total cell count to 1.5 × 10^3^ ± 0.5 × 10^3^ cells mL^−1^ in water leaving the treatment plant. This removed a limitation of both flow cytometry and 16S rRNA amplicon sequencing, which have difficulties in resolving small changes against a high background cell count. Following installation, 58% of the bacteria in the distributed water originated from the pipe biofilm, in contrast to before, when 99.5% of the cells originated from the treatment plant, showing that UF shifts the origin of the drinking water microbiome. The number of bacteria released from the biofilm into the distributed water was 2.1 × 10^3^ ± 1.3 × 10^3^ cells mL^−1^ and the percentage of HNA (high nucleic acid) content bacteria and intact cells increased as it moved through the distribution system. DESeq2 analysis of 16S rRNA amplicon reads showed increases in 29 operational taxonomic units (OTUs), including genera identified as *Sphingomonas*, *Nitrospira*, *Mycobacterium*, and *Hyphomicrobium*. This study demonstrated that, due to the installation of UF, the bacteria entering a drinking water microbiome from a pipe biofilm could be both quantitated and described.

## Introduction

Drinking water is delivered to the consumer through kilometres of pipes and maintenance of water quality in these drinking water distribution systems (DWDSs) is a prime concern for drinking water providers. These systems contain microorganisms in both the flowing water and in biofilm that lines the interior of the pipes.^1,2^ This pipe biofilm may: be a reservoir for pathogens^3,4^; play a role in corrosion^5^; and, impact the aesthetics of the water.^6^

The complex microbial communities of DWDS biofilms^7,8^ are distinct from that of the bulk water and differ according to water and location. Bacteria in loose deposits and pipe biofilm were estimated to contain >98% of the bacteria in a DWDS^7^ and release of these cells can alter the bulk water.^8^ This must always be occurring to some extent,^9^ although most studies have focussed on large changes due to season, water pressure or flow in the microbial communities in the biofilm or distributed water.^10–12^ Changes in microbial communities in distributed water associated with increasing distance have been attributed to spatial dynamics, including disinfection residuals and pipe connections, and pipe biofilm was suggested as a source of this variation.^13^ Liu and colleagues, however, estimated that the majority of bacteria in tap water originated from the treatment plant, with cells from the biofilm contributing only a few percent.^14^

These studies characterized DWDS biofilm material detached by flushing or swabbed from surfaces, and interactions of pipe biofilm with distributed water during normal hydraulic operating conditions have been difficult to observe.^3^ This is likely due to the small number of bacteria entering from the biofilm, relative to the number of cells present in the distributed water, which also limits the application of analysis methods for bacterial communities. 16S rRNA gene amplicon sequencing cannot resolve bacteria at very low abundance, against a high abundance background community^15^; and flow cytometry (FCM) cannot detect changes representing <5% of the total cell count.^16^

The drinking water treatment plant (DWTP) in Varberg, Sweden was upgraded to include a full-scale ultrafiltration (UF) facility with two-stage filtration and in-line coagulation at the primary membrane stage.^17^ This change created a full-scale DWDS that distributed water containing altered natural organic matter (NOM) and virtually no bacteria. Changes in biofilm likely require extended time frames to respond to a new environment,^18^ so the days immediately following UF installation provided a window of opportunity before changes in the water quality would impact the biofilm. With fewer bacterial cells in the distributed water, those originating from the pipe biofilm and released into the water could now be observed. Sampling locations were chosen with short water retention times to ensure that cells detected in the water phase could not be the result of regrowth. The removal of the high background cell count removed the limitations in resolution for FCM and 16S rRNA amplicon sequencing studies and the community of bacteria released from the pipe biofilm in a full-scale DWDS could be quantitated and described, as they shaped the post-UF drinking water microbiome.

## Results

### Impact of UF installation

**Fig. 1:**
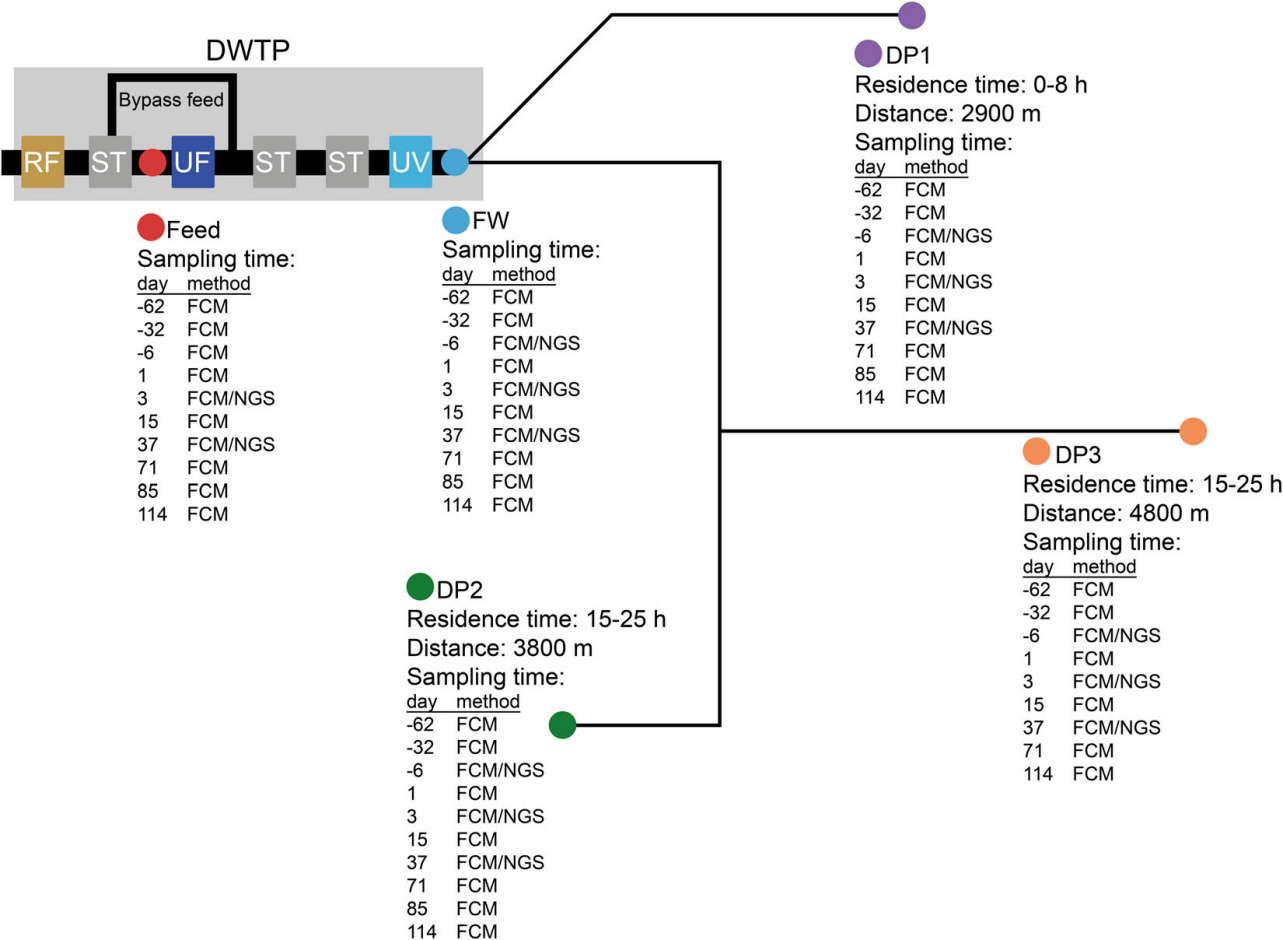
Schematic illustration of the treatment plant process and sampling points in the distribution network. Locations of DP1, 2 and 3 are not to scale. Distance and time for the water to reach the sampling location from the treatment plant, and types of samples taken on each day, are indicated. In the first 37 days after UF installation, a small fraction of water bypassed the filter, and was used for pH adjustment. After day 37, pH was adjusted using water from UF permeate. *RF* rapid sand filter, *ST* storage tank, *DWTP* drinking water treatment plant

**Fig. 2:**
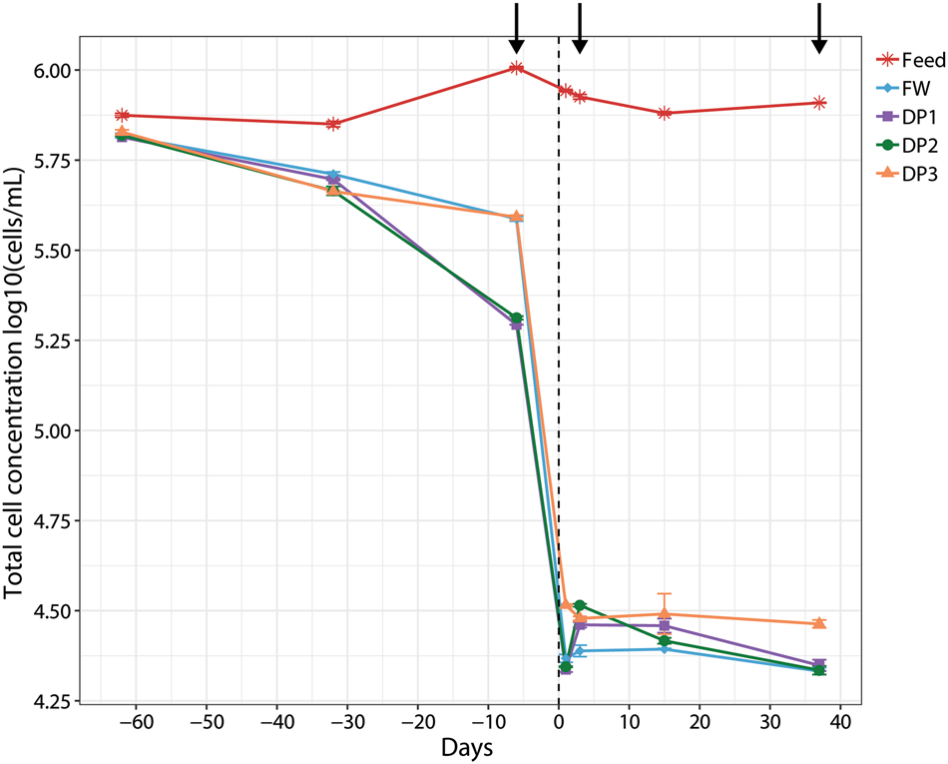
The number of bacteria in water from the treatment plant and distribution system in the first 37 days following UF installation. TCC were measured in the feed water to the UF (red line, stars); water leaving the treatment plant (finished water FW, blue line, diamonds); and at DP1 (purple line, squares), DP2 (green line, circles) and DP3 (orange line, triangles) in the distribution system, before and after the installation of UF. Day 0 on the *x* axis corresponds to the start of UF (vertical dashed line). The arrows indicate days when water was sampled for sequencing. Error bars represent the variation in technical triplicates

Bacteria in water samples from the DWTP (feed, finished water) and the DWDS (distributed water) were quantified and described by FCM before, and in two distinct time periods after, installation of UF (Fig. 1). From day 0 to day 37, water from the UF feed, and thus containing bacteria, was used for pH regulation, resulting in the addition of approximately 2.2 × 10^4^ ± 4.5 × 10^2^ cells mL^−1^ to the UF permeate, whereas after day 37, and until the end of the study period, pH was adjusted using only UF permeate. The permeate had a total cell concentration (TCC) below the quantification limit (data not shown), at around 200 cells mL^−1^,^16^ and this was reflected by the instantaneous reduction in TCC at all distribution points (DP), and time points sampled after UF installation (Fig. 2).

In the first 37 days after UF installation, the average TCC of distributed water samples decreased from 4.8 × 10^5^ ± 1.7 × 10^5^ cells mL^−1^ (*n* = 27) to 2.7 × 10^4^ ± 4.3 × 10^3^ cells mL^−1^ (*n* = 36), a reduction of 93.1 ± 3.3%. This TCC in the distributed water included bacteria released from pipe biofilm and those added during the pH adjustment. Before UF installation, the concentration of cells with high nucleic acid (HNA) content did not change during distribution (48 ± 7.8% finished water; 48 ± 7.5% distributed water). However, in the 37 days after UF installation, the proportion of HNA content bacteria in distributed water increased significantly (*P* < 0.01, one-way analysis of variance (ANOVA)) (Supplementary Figure 1): from 39 ± 2.3% in the finished water to an average of: 40 ± 3.2% at DP1, 44 ± 4.5% at DP2, and 43 ± 3.6% at DP3. During this initial period, intact cell concentration (ICC) in distributed water decreased, from an average of 58 ± 6.0% to 26 ± 6.1% (Supplementary Figure 2) although ICC increased in distributed water compared to finished water, from 19 ± 3.8% to 26 ± 6.1%. This was not observed before the installation of UF (55 ± 3.3% ICC and 58 ± 6.0% ICC, respectively).

Conventional water quality parameters were measured before, and 3 and 37 days after, installation of UF (Supplementary Table 2). Colour, dissolved organic carbon (DOC), turbidity and total organic carbon (TOC) decreased in finished and distributed water after UF installation. Heterotrophic bacteria were only observed after 7 days of incubation and increased slightly in distributed water compared to finished water, regardless of UF treatment. At DP2, water temperature was always highest and took longer to stabilize, nitrite concentrations were lowest and copper concentrations were highest. UF installation also altered NOM (Supplementary Table 3).

### Phylogenetic analysis of the bacterial communities

**Fig. 3:**
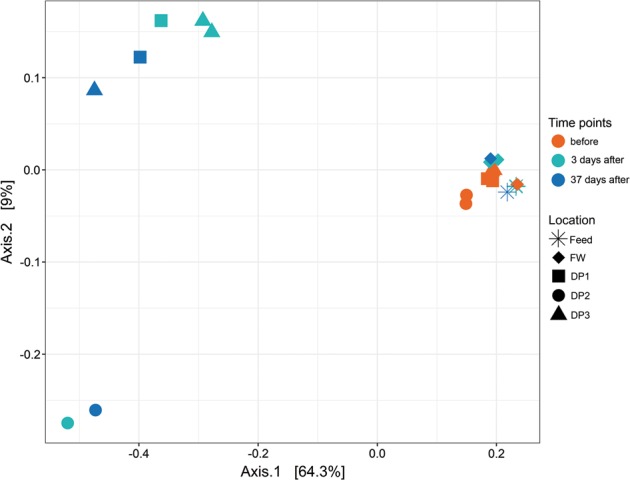
Comparison of bacterial communities before, and in the first 37 days after, installation of UF using a principal coordinates analysis (PCoA) plot based on Bray–Curtis dissimilarity calculated for bacterial communities from water sampled in the treatment plant: at feed (stars) and finished water (FW, diamonds); and at DP1 (squares), DP2 (circles) and DP3 (triangles). Samples were taken before installation of UF (orange) and at 3 (green) and 37 (blue) days after installation. Communities associated with the distribution system after installation of UF were separated from all other samples

The UF installation did not appear to alter the community composition in the finished water (Fig. 3); however, in the first 37 days, pre-UF water (feed) was used to adjust pH, so this community was in fact a dilution of the feed water community with UF permeate and would have little impact on comparisons based on relative abundance. In contrast, after installation, relative abundance of *Alphaproteobacteria* and *Nitrospira* significantly increased (*P* < 0.05, Mann–Whitney *U* test) in water from the DWDS (Supplementary Figure 3). The average relative abundance of *Alphaproteobacteria* in distributed water was 20 ± 1.9% before UF (*n* = 6), with limited variation between DPs, while after installation, *Alphaproteobacteria* increased in relative abundance at DP1 (42 ± 5.9%, *n* = 2), DP2 (36 ± 7.7%, *n* = 2) and DP3 (35 ± 1.7%, *n* = 3). Increased relative abundance of *Nitrospira* was observed at DP1 (from 0.87 ± 0.061% to 5.9 ± 0.29%) and DP3 (from 0.97 ± 0.061% to 11 ± 7.6%) with the largest change seen at DP2, from 3.2 ± 0.44% before UF to the highest observed relative abundance for this class, at 30 ± 11%, after the installation.

**Fig. 4:**
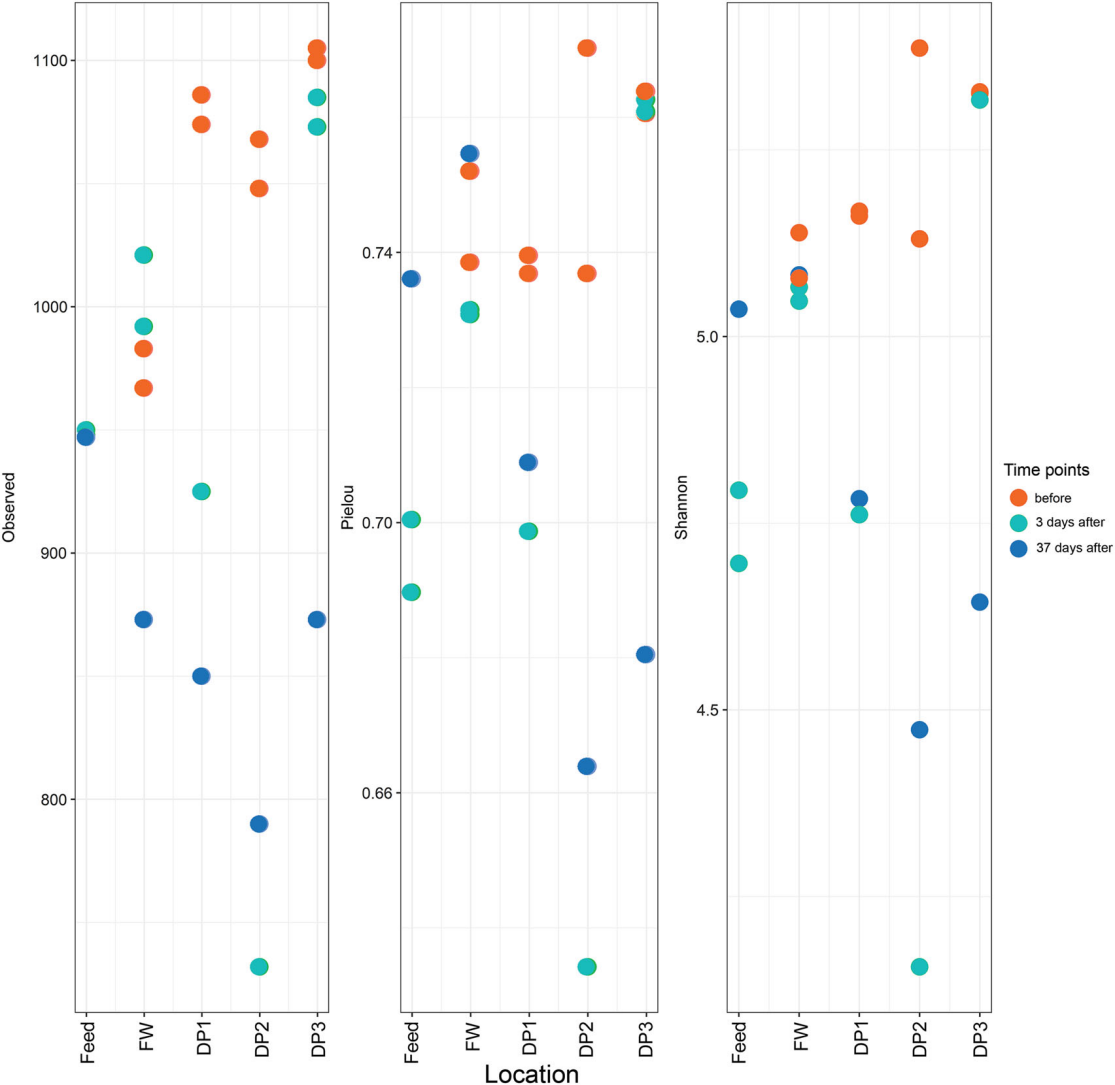
Installation of UF impacts diversity of bacterial communities in the distributed water. Alpha diversity analysis of bacterial communities in water samples from the treatment plant (feed and finished water (FW)) and distribution system (DP1, 2, 3) were examined before installation of UF (orange) and at 3 (green) and 37 (blue) days after installation. The number of observed OTUs (left), evenness (Pielou’s index) (middle) and Shannon index (right) are compared in the different communities

Communities at different locations within the DWDS diverged from those in post-UF installation finished water (feed water diluted in permeate) and all of the communities before UF installation (Fig. 3). Communities at DP1 and DP3 were most similar, whereas those at DP2 had a distinct composition. Bacterial communities in the distributed water from before the installation of UF showed highest richness (number of operational taxonomic units (OTUs), sequence similarity cut-off: 97%) and evenness (Pielou’s index) and thus the highest diversity (Shannon index, Fig. 4). UF installation did not affect diversity in the finished water, due to the dilution with feed water (Shannon index, 5.1 ± 0.018 vs. 5.1 ± 0.043); however, communities in distributed water had larger variation and significantly lower diversity (*P* < 0.05, one-way ANOVA) after the installation. The community at DP2, 3 days after installation, contained the fewest OTUs (732) and lowest evenness (0.63). All rarefaction curves reached a plateau (Supplementary Figure 4).

### Identifying the bacteria released from the biofilm

**Fig. 5:**
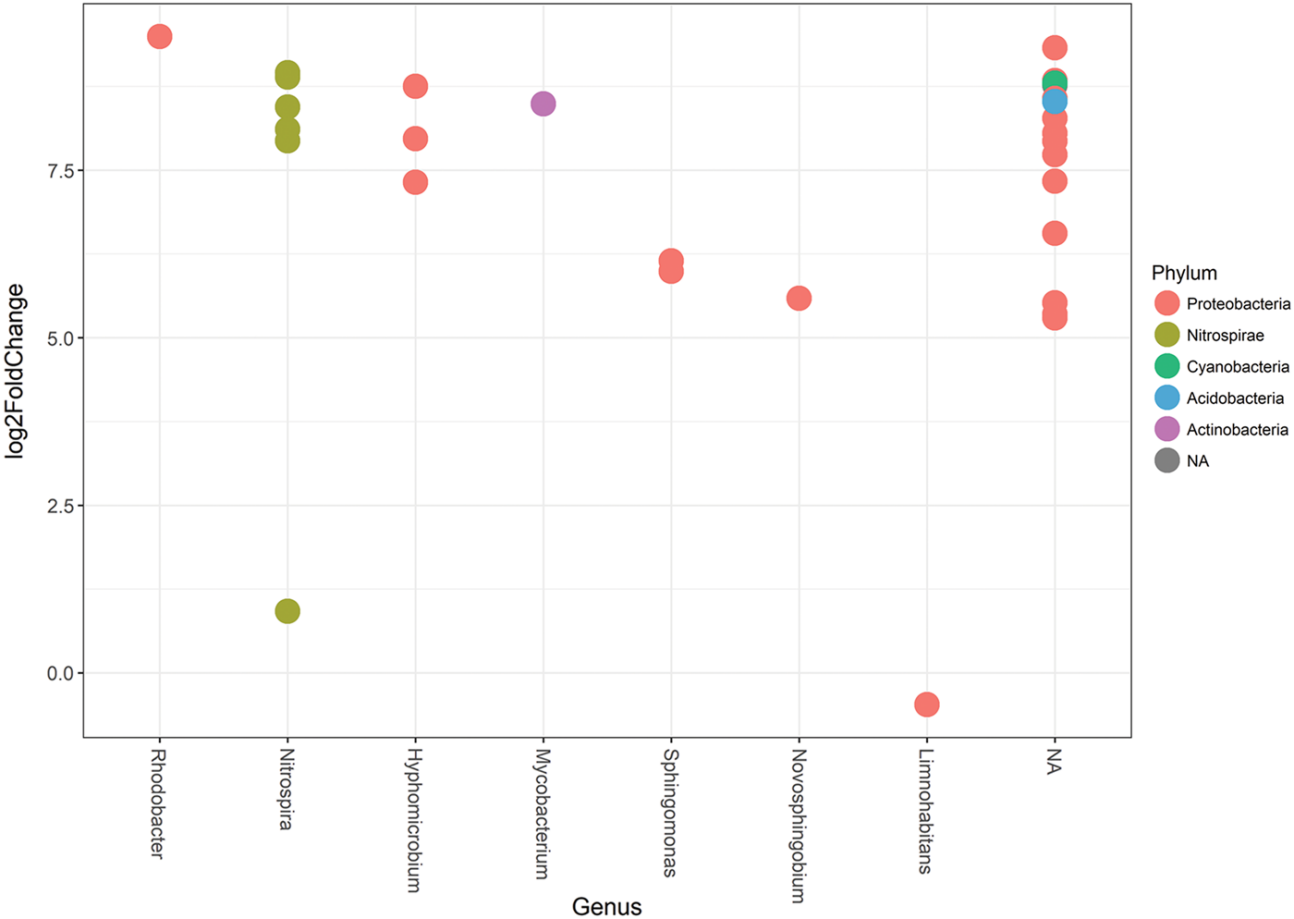
Operational taxonomic units (OTUs) representing changes in the bacterial community of the distributed water. Log2 fold changes calculated by DESeq2 in R for OTUs describe changes in the bacterial community in the distributed water after the installation of ultrafiltration. Each dot represents an OTU with the classified taxonomic level (genus) shown on the *x* axis, and phylum indicated by colour. A positive value indicates a significant increase of the specific OTU in the distributed water community relative to that of the finished water leaving the treatment plant

After installation of UF, sequencing reads originating from finished water were compared to those from distributed water using DESeq2 analysis, with 147 OTUs containing at least 0.1% of the total unrarefied number of sequences as input. Thirty OTUs with a significant change in number of reads (*P* < 0.01) were identified (Supplementary Table 1) with 15 of these classified into 7 genera (Fig. 5). Reads increased >300-fold in distributed water for OTUs classified as *Rhodobacter* (1 OTU), *Nitrospira* (3 OTUs)*, Hyphomicrobium* (1 OTU) and *Mycobacterium* (1 OTU) and >150-fold for 2 OTUs classified as *Nitrospira* and *Hyphomicrobium*. OTUs where reads increased >30-fold were classified as *Sphingomonas* (2 OTUs) and *Novosphingobium* (1 OTU). A 30-fold increase was also observed in 14 additional OTUs classified at the family, order and class level (Fig. 5). One OTU could not be classified.

**Fig. 6:**
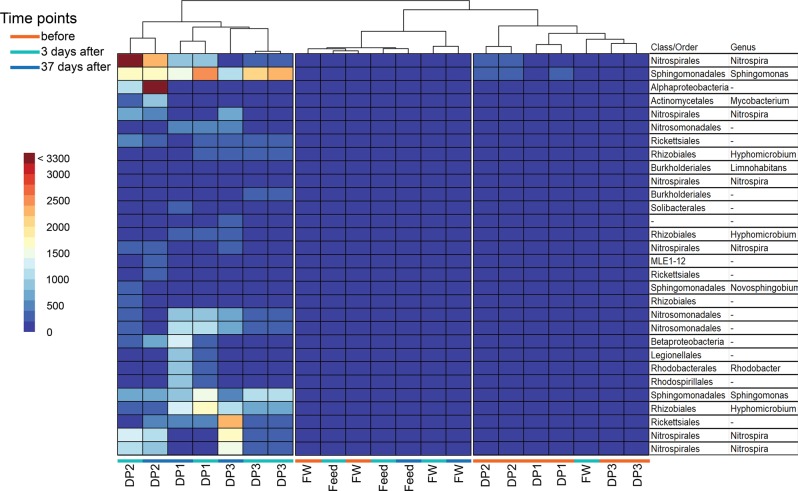
Changes in read frequencies for specific OTUs at different sampling points in the distribution system. The heatmap shows frequency of reads in the 30 OTUs selected by DESeq2 analysis for feed and finished water (FW) from the treatment plant and DP1, 2 and 3 before installation of ultrafiltration (orange) and at 3 (green) and 37 (blue) days after installation. The classification of the OTUs in class/order and genus are shown to the right of the figure

To examine local variations, the abundance of reads within OTUs selected by DESeq2 were compared at each DWDS sampling point, before and after the installation of UF (Fig. 6). This separated DWDS samples collected after the UF installation from all other samples in the study. One OTU classified as *Nitrospira* accounted for 4.5% (5595 of the 24,900 reads) of the total rarefied reads from DP2 sampled 3 days after installation of UF. Communities sampled at DP1 and DP3 after the installation of UF had higher relative abundance for an OTU belonging to the genus *Sphingomonas* (1980 ± 820 reads and 1860 ± 630 reads, respectively) compared to the period before (175 ± 69 reads and 158 ± 59 reads, respectively). A high relative abundance of *Rickettsiales* (2264 reads) and increases in two OTUs belonging to *Nitrospira* were also observed at DP3, on day 37.

### Quantifying the bacteria released from the biofilm

After day 37, feed water was replaced with UF permeate for pH adjustment (Supplementary Figure 5), further minimizing the number of cells in the finished water leaving the DWTP. This exposed changes in TCC that could be attributed to release of cells from the biofilm. Finished water now contained an average of 1.5 × 10^3^ ± 0.5 × 10^3^ cells mL^−1^ (*n* = 9) from UF permeate (approximately 200 cells mL^−1^) and contact with biofilms within the treatment plant (Fig. 1). With the average TCC of the distributed water after day 37 of 3.7 × 10^3^ ± 1.2 × 10^3^ cells mL^−1^ (*n* = 24, Supplementary Figure 5), the release of bacterial cells from the DWDS biofilm was an average of 2.1 × 10^3^ ± 1.3 × 10^3^ cells mL^−1^ or approximately 58% of the TCC in the water. The numbers of cells increased with increasing distance from the treatment plant: with 47% at DP1 (*n* = 9), 60% at DP2 (*n* = 9), and 65% at DP3 (*n* = 6) with similar trends observed in the proportion of cells with HNA (from 38% ± 12% to 59% ± 6.9%) and ICC (from 46% ± 18% to 60% ± 10%). Using the estimate of bacterial release from the pipe biofilm (2.1 × 10^3^ ± 1.3 × 10^3^ cells mL^−1^) with the average TCC of distributed water *before* the UF installation (4.8 × 10^5^ ± 1.7 × 10^5^ cells mL^−1^), the percentage of bacteria from the biofilm in the bacterial population of this distributed water was estimated at 0.5%, with 99.5% originating from within the treatment plant. Taken together, this shows that UF installation shifted the bacterial community in the distributed water so that, with increasing distance from the DWTP, it was increasingly comprised of bacteria released from pipe biofilm.

## Discussion

Ultrafiltration impacts many aspects of water quality, including changes in the amount and character of both NOM and bacteria.^19^ The installation of UF reduced the TCC in the distributed water from 4.8 × 10^5^ ± 1.7 × 10^5^ cells mL^−1^ to 3.7 × 10^3^ ± 1.2 × 10^3^ cells mL^−1^, corresponding to a 99% removal of bacteria in the DWDS. This degree of cell removal exposed small relative differences between water sampled at different points within the distribution system, permitting quantification and identification of bacteria from the pipe biofilm that were released into the water as it travelled through the distribution system.

While studies have suggested that the microbiome in distributed drinking water is highly influenced by biofilm on pipe walls,^1,20^ others have contradicted this hypothesis^7,21^ and suggested that source water^10,22,23^ and sand filters^24,25^ are more influential. In the current study, after day 37, 58% of the bacteria in the distributed water originated from pipe biofilm. While one explanation for this addition of cells to the water could be regrowth, the DWDS sampling points in this study had short residence times (>25 h), and with a growth rate approximated as 0.30 day^−^^1^ (or a doubling time of 2.31 days) for distributed water,^26^ this is an unlikely explanation for the increases in TCC. Nutrient concentrations (DOC, biopolymers, and humic substances, Supplementary Table 3) were reduced by UF; water temperatures ranged from 5.7 to 9 °C (Supplementary Table 2); and 7-day incubation were required to detect heterotrophs (Supplementary Table 2). Taken together, this evidence strongly suggests that the increase in TCC with distance from the treatment plant was due to release of cells from the pipe biofilm into the water.

The short time frame in this study allowed the contribution of cells from the biofilm to be estimated as 0.5% of the total cells present in the water *before* the change. Applying this estimate for cells released from the biofilm to other systems where the bacterial concentration in the distributed water is high can explain why the contribution from the pipe biofilm to the water microbiome has been difficult to observe. In a year-long sampling campaign by Pinto and colleagues (2014), only water sampled at great distance from the DWTP showed small changes in the water microbiome.^27^ Henne and colleagues (2012) compared communities from distributed water and biofilm, and the water had a highly homogeneous bacterial community despite observed diversity in the biofilm communities.^8^ We suggest that the community composition in the distributed water will be clearly associated with processes in the treatment plant, such as the use of sand filters^24,25,28^ and use of disinfectants^29–31^ unless that treatment (i.e. UF) removes a large percentage of cells. In this case, the bacterial community in the distributed water will contain a majority of cells originating from the pipe biofilm. Given the great diversity in the microbial communities of source water, distributed water and biofilm and other variables governing water quality such as local climate, treatment processes and pipe materials, it is not known if the bacterial community in this study, and the extent to which it was released into the flowing water, reflects what would happen in every DWDS, and additional studies are needed to determine the impact of UF in other systems.

After installation of UF, the percentage of HNA bacteria in the distributed water increased compared to finished water (Supplementary Figure 1). Proctor and colleagues (2018) proposed that HNA bacteria, in contrast to low nucleic acid bacteria, are not as dependent on other bacteria for survival^32^ and HNA bacteria may survive in distributed water without the biofilm community. The percentage of intact cells also increased in the water as it travelled through the DWDS, and may be a signature for bacterial release from pipe biofilm. Shifts in HNA^33^ and ICC^34^ were observed in tap water after overnight stagnation and distributed water, respectively, and may indicate release of biofilm in these contexts.

DNA sequencing studies of bacterial communities in pipe biofilms have shown higher diversity compared to that in the distributed water.^8,35^ Henne and colleagues (2012) showed higher diversity with lower richness in the biofilm compared to the water phase and suggested that the biofilm community contains evenly distributed members adapted for this specific environment.^8^ This implies that if only some members of the evenly distributed biofilm community are released into the distributed water there will be a shift in the population towards lower evenness. In the current study, lower diversity (due to both decreased richness and lower evenness) was observed for the community in distributed water after UF installation, compared to those in finished water and before UF installation. This altered community structure in the distributed water can be attributed to interaction with the biofilm, with the similarity between the communities in finished water and before UF installation attributed to the use of diluting feed water for pH adjustment in the first 37 days after UF installation. In this period, lower evenness was observed as increasing dominance in the distributed water of a few specific OTUs, such as genera *Nitrospira*, and *Sphingomonas*. Lower diversity in the water microbiome has been observed after flushing, with this uneven detachment of biofilm resulting in a more uneven water community.^12,36^

Installation of UF decreased the richness (lower numbers of OTUs) in the distributed water. A rich bacterial community in the water, with many bacteria at low abundance, can be a seed bank for the biofilm community.^8^ Altered environmental conditions initiated by the UF treatment could trigger cells to enter the biofilm, resulting in the observed decrease in richness.^37^ This would not appear in the DESeq2 analysis, as this only included OTUs with total read abundance across all the samples >0.1%, and it has been suggested that the rare biosphere represented by OTUs with abundance <0.1% of the community is the dormant microbial seedbank.^37^

Specific OTUs at class level accounted for much of the observed changes in the water microbiome, including *Alphaproteobacteria* and *Nitrospira*, which showed a higher relative abundance in the distributed water after the installation of UF. Higher relative abundance of *Alphaproteobacteria* has been observed in biofilm compared to the distributed water,^8^ in water containing biofilm detached by flushing^12^ and dominating biofilm communities in DWDS^7^ and water meters.^38,39^ Observations similar to these seen for *Alphaproteobacteria* have also been observed for *Nitrospira*.^21,35^

Bacteria released from the biofilm were described by 29 OTUs where the absolute read abundance increased in the distributed water compared to the finished water. Two OTUs classified as genus *Sphingomonas* predominated at DP1 and DP3 relative to DP2 and compared to the rest of the OTUs describing the released biofilm community. *Sphingomonas* are often detected in bacterial communities from drinking water, with high abundance in biofilms^10,38^ and a relative abundance in DWDS estimated at up to 85%.^7^ *Sphingomonas* possess flagella,^40^ with this motility perhaps contributing to their release from the biofilm and their proposed role as early colonizers DWDS biofilms.^41^ *Sphingomonadaceae* are HNA bacteria (as large bacteria >0.4 µm),^32^ which supports observed increase of HNA bacteria in distributed water in the current study.

Six of the 29 OTUs released from the biofilm were classified as genus *Nitrospira*, a group of bacteria that has been found in bacterial communities in drinking water, loose deposits and drinking water biofilms.^7,8,21,35^ The dominance of this taxa at DP2 might be due to loose deposits containing high amount of biofilm with *Nitrospira* abundance, which can vary between locations in the distribution, although this was not examined in the current study. Members of this genus can use nitrite as an electron donor instead of organic molecules^21,42^: nitrite concentrations at DP2 were lower compared to DP1 and DP3. DP2 was consistently warmer, with higher copper concentrations and low-carbon, chloramine-treated water, which may also favour growth of *Nitrospira*.^43^

While numerous studies have associated *Alphaproteobacteria*, *Sphingomonas*, *Nitrospira* and *Mycobacterium* spp. with drinking water and its biofilms, this study showed that members of these classes and genera also move from the pipe biofilm into the drinking water. It does not appear to be a single mode of motility that is used to escape the biofilm: *Sphingomonas* are almost universally motile via flagella; *Nitrospira* are generally thought to be nonmotile,^44^ *Mycobacterium spp*. use sliding motility^45^ and *Hyphomicrobium* are motile as swarmer cells with flagella.^46^ All modes may be sufficient and, together with random attachment and detachment, account for cell release.^9^ This could occur for both live and dead cells leaving the biofilm, and it would be interesting to describe this community in the context of cell viability.

In conclusion, although the UF installation modified the type of organic matter and greatly reduced the number of bacterial cells in the distributed water, destabilization of the biofilm, observed as detachment, sloughing or a sudden increases in the number of total cells in distributed water, was not observed during the 114 days of the study. It can take years for changes to be observed in a microbial community in response to an alteration in the environment,^47^ so the observation of consistently low cell counts over the 0.3 year of the current study does not confirm that this will always be the case and it is not known how this biofilm will adapt over the coming years and seasons to the UF installation. Regions in the DWDS with longer retention times may gradually show increasing cell counts in distributed water from prolonged contact with the biofilm or the dynamics of bacterial release may change. Changes in nutrients, such as those described in this study (both NOM and cells), may, over months and years, change the water and biofilm community composition as they adapt to these new conditions.^18^ Since this study was conducted during winter, it is also not known to what extent the release of bacterial cells could change with increases in temperature or seasonal changes in water use, which have both been shown to alter the overall numbers of cells in distributed water.^34^ The impact of having a higher percentage of bacteria in the water that originates from biofilm is also not known. Given that cells originating from biofilms are more likely to form biofilms themselves,^48^ it would be interesting to see whether shifts in the origin of the bacteria in the distributed water can impact formation of biofilms on new DWDS pipes, water meters or household drinking water plumbing systems.

## Methods

### Study site and sampling

Water samples were collected from Kvarnagården Waterworks and DWDS operated by VIVAB (Varberg, Sweden). Treatment consisted of pH adjustment, rapid sand filtration, ultraviolet (UV) disinfection and distributed with a chlorine residual. In November 2016, the DWTP was reconfigured to use rapid sand filtration, coagulation and UF, pH adjustment, UV disinfection and chlorine residual between 0.13 and 0.21 mg L^−1^. For the first 37 days following UF installation, UF feed water was used for pH adjustment of the finished water, then switched to use UF permeate. Feed water refers to water sampled after rapid filters, after the UF installation. After the addition of chloramine, the water is referred to as finished.

The approximate location, distances and residence times describing the DWDS sampling locations (DP1–3, Fig. 1) were provided by the water company. DP1 is an office building tap at a wastewater treatment plant (VIVAB), DP2 and DP3 are sampling taps at a school and pump station, respectively. Water samples for FCM were collected in sterile 15 mL Falcon tubes, stored on ice or at 4 °C and analysed the following day. Chlorine residuals were quenched by addition of 1% (v/v) sodium thiosulphate (20 g L^−1^). Water samples for sequencing analysis (1 L for before UF installation, and feed water, 5 L for after UF installation) were collected in sterilized borosilicate bottles, filtered onto 0.22-µm filters (Merck, Germany), stored on ice during travel to the laboratory and at −20 °C until DNA extraction. Conventional water quality sampling and analysis was according to the analysis laboratory Eurofins Scientific (Belgium). NOM was analysed by LC-OCD-OND at DOC-Labor (Germany).

### FCM analysis

FCM analysis was performed according to Prest et al.^49^ using a BD Accuri C6 flow cytometer (Becton Dickinson, Belgium) equipped with a 50 mW laser, emission wavelength at 488 nm. Briefly, water samples in triplicate were stained with 5 µL mL^−1^ of SYBR Green I at 100× diluted with dimethyl sulphoxide (stock concentration 10,000 × , Invitrogen AG, Switzerland) at room temperature to a final concentration of 1× SYBR Green I and incubated at 37 °C for 15 min. ICC was determined by including 3 µM propidium iodide (1 mg mL^−1^, Sigma-Aldrich, Germany). Stained samples (50 µL of the 500 µL) were analysed with a threshold of 500 arbitrary units of green fluorescence. Results were exported as FCS files to FlowJo (Tree Star Inc, USA) and gated identically for all samples with green fluorescence (533 ± 30 nm) and red fluorescence (>670 nm). The number of HNA bacteria were determined using a cut-off for green fluorescence >2 × 10^4^ arbitrary units.^49^ One-way ANOVA tests were conducted in R.^50^

### Microbial community analysis

DNA was extracted using the Fast DNA Spin Kit for Soil according to the manufacturer’s instructions from filter papers cut into strips and added directly to tubes containing Lysing Matrix E (MP Biomedicals, USA). Empty filter papers were extracted as a negative control. Extracted DNA was stored at −20 °C until further processing.

Amplicons of the V3–V4 region of 16S rRNA gene were generated using the universal bacterial primers Bact_341F (5´-CCTACGGGNGGCWGCAG-3´) and Bact_785R (5´-GACTACHVGGGTATCTAATCC-3´).^51^ PCR reactions (25 µL) containing: 12.2 µL Milli-Q water, 10 µL 5PRIME HotMasterMix (Quantabio, USA), 0.8 µL (10 mg mL^−1^) bovine serum albumin, 0.5 µL (10 µM) of forward and reverse primers, and 1 µL of template DNA were cycled for 94 °C for 3 min followed by 35 cycles of: 94 °C for 45 s, 50 °C for 1 min, 72 °C for 1.5 min, and a final step of 72 °C for 10 min. Three PCR reactions were performed for each DNA extraction, triplicates were combined and each amplicon was quantified using a Qubit 2.0 dsDNA BR Assay Kit (Thermo Fisher Scientific, USA). Amplicons were inspected by agarose gel electrophoresis, and as sufficient DNA was obtained, no additional measures were required in order to proceed, regardless of the initial volume of water sampled (1 L, 5 L). DNA from each amplicon (50 ng) were then pooled together, purified using the UltraClean PCR Clean-up Kit (Qiagen, Germany) according to the manufacturer’s instructions) and quantified again using Qubit. Sequencing was performed on the MiSeq platform using the MiSeq Reagent Kit v3 (600-cycles) (Illumina, USA), according to the manufacturer’s instructions, with 10% PhiX added to the sequencing run.

Sequencing data was analysed using Quantitative Insights into Microbial Ecology (QIIME) pipeline.^52^ OTUs were clustered with 97% sequence similarity using the open reference OTU-picking method in QIIME. Chimeras were identified using the UCHIME algorithm^53^ integrated in the USEARCH^54^ pipeline. Taxonomy assignments and sequence alignments with the PyNAST alignment were performed using the GreenGenes database. Analysis using the OTU table (biom format) was performed in R using the phyloseq package,^55^ displayed by ggplot2 package.^56^ OTUs with total reads across all samples <0.005% were removed and the library rarefied to 24,900 reads per sample. The negative control was removed from further analysis as the number of reads in these samples was lower than the rarefied threshold. Alpha diversity was calculated using Shannon index for diversity by the vegan package^57^ and Pielou’s index for evenness using the function *evenness* from the microbiome package^58^ in R. The Principal Coordinates Analysis (PCoA) plot was created using the Bray–Curtis dissimilarity matrix from the vegan package. Clusters formed in the PCoA plot could not be confirmed by permutational analyses of variance due to uneven dispersion in the data set (tested by the function *betadisp* in R, vegan package).

OTUs with differential read abundance were identified using the DESeq2 package^59^ in R. OTUs with total unrarefied reads across all samples >0.1% were used as input with distributed water samples collected after UF as one group compared to the finished water samples after UF. The OTUs selected by DESeq2 analysis were used to construct a heatmap using the pheatmap package^60^ in R.

### Reporting Summary

Further information on experimental design is available in the Nature Research Reporting Summary linked to this article.

## Supplementary information

Supplementary Information - Figures and Tables

Reporting Summary

## Data Availability

DNA sequences are available at the NCBI Sequence Read Archive, accession number PRJNA494637.

## Supplementary information

**Supplementary Information** accompanies the paper on the *npj Biofilms and Microbiomes* website (10.1038/s41522-019-0082-9).
