# Supplementary material for: Bacterial release from pipe biofilm in a full-scale drinking water distribution system
Source: NPJ Biofilms Microbiomes. 2019 Feb 22;5:9. doi: 10.1038/s41522-019-0082-9 (PMC6385293; doi:10.1038/s41522-019-0082-9)
Supplement: Supplementary file 1 — Supplementary Information - Figures and Tables [file 41522_2019_82_MOESM1_ESM.pdf]

Supplementary Information - Figures

Bacterial release from pipe biofilm in a full-scale drinking water distribution system

Sandy Chan[a,b,c,], Kristjan Pullerits[a,b,c], Alexander Keucken[d,e], Kenneth M. Persson[b,c,d], Catherine J. Paul[a,d*], Peter Rådström[a]

[a]Applied Microbiology, Department of Chemistry, Lund University, P.O. Box 124, SE-221 00 Lund, Sweden

[b]Sweden Water Research AB, Ideon Science Park, Scheelevägen 15, SE-223 70 Lund, Sweden

[c]Sydvatten AB, Hyllie Stationstorg 21, SE-215 32 Malmö, Sweden

[d]Water Resources Engineering, Department of Building and Environmental Technology, Lund University, P.O. Box 118, SE-221 00 Lund, Sweden

[e] Vatten & Miljö i Väst AB, Box 110, 311 22 Falkenberg, Sweden

*****corresponding author*

<u>Table of Contents</u>

Supplementary Figure 1 – Changes in high nucleic acid (HNA) content bacteria in DWDS

Supplementary Figure 2 – Intact cell concentrations in the DWTP and DWDS

Supplementary Figure 3 – Relative abundance of bacteria in the different samples at the class level

Supplementary Figure 4 – Rarefraction curves

Supplementary Figure 5 – TCC of the distributed water samples after UF installation

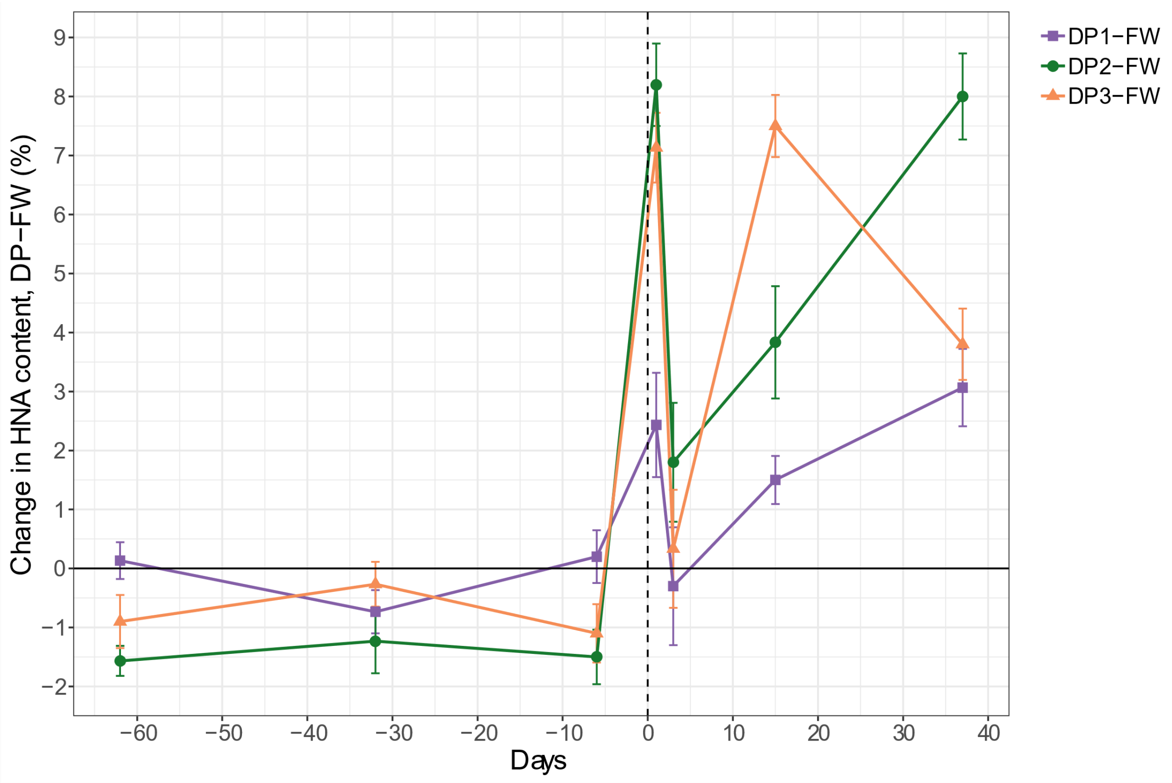

Supplementary Figure 1 – Changes in high nucleic acid (HNA) content bacteria showing an increase in % HNA in distributed water following installation of UF. For each sample, the %HNA in the finished water was subtracted from that measured at DP1 (purple line, squares), DP2 (green line, circles) and DP3 (orange line, triangles) in the distribution system, before and after the installation of UF. Day 0 on the x-axis corresponds to the start of UF (vertical dashed line). Error bars represent the variation in technical triplicates.

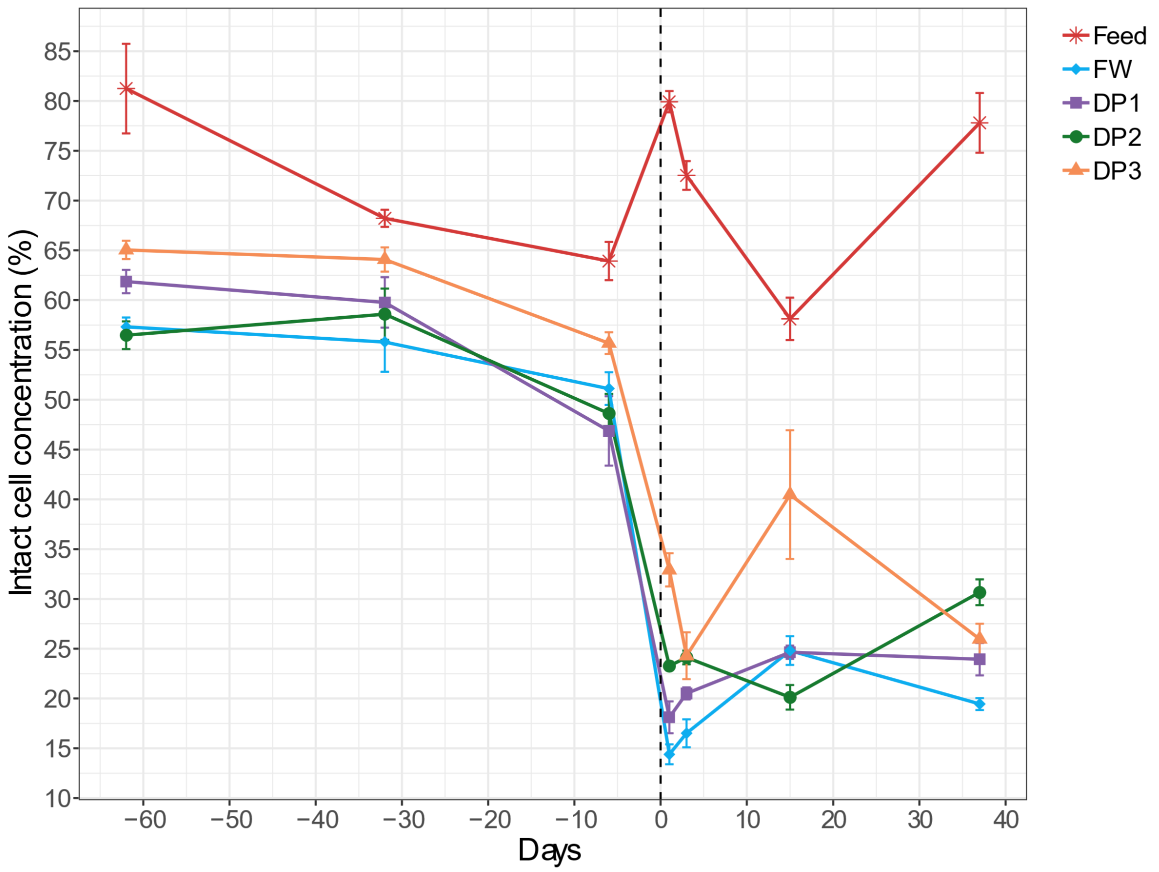

Supplementary Figure 2 – Intact cell concentration measured at the treatment plant for feed water to the UF (red line, stars), finished water (FW, blue line, diamonds), and at DP1 (purple line, squares), DP2 (green line, circles) and DP3 (orange line, triangles) in the distribution system, before and after the installation of UF. The days on the x-axis corresponds to a start of ultrafiltration at day 0 (vertical dashed line). Error bars represent the variation in technical triplicates.

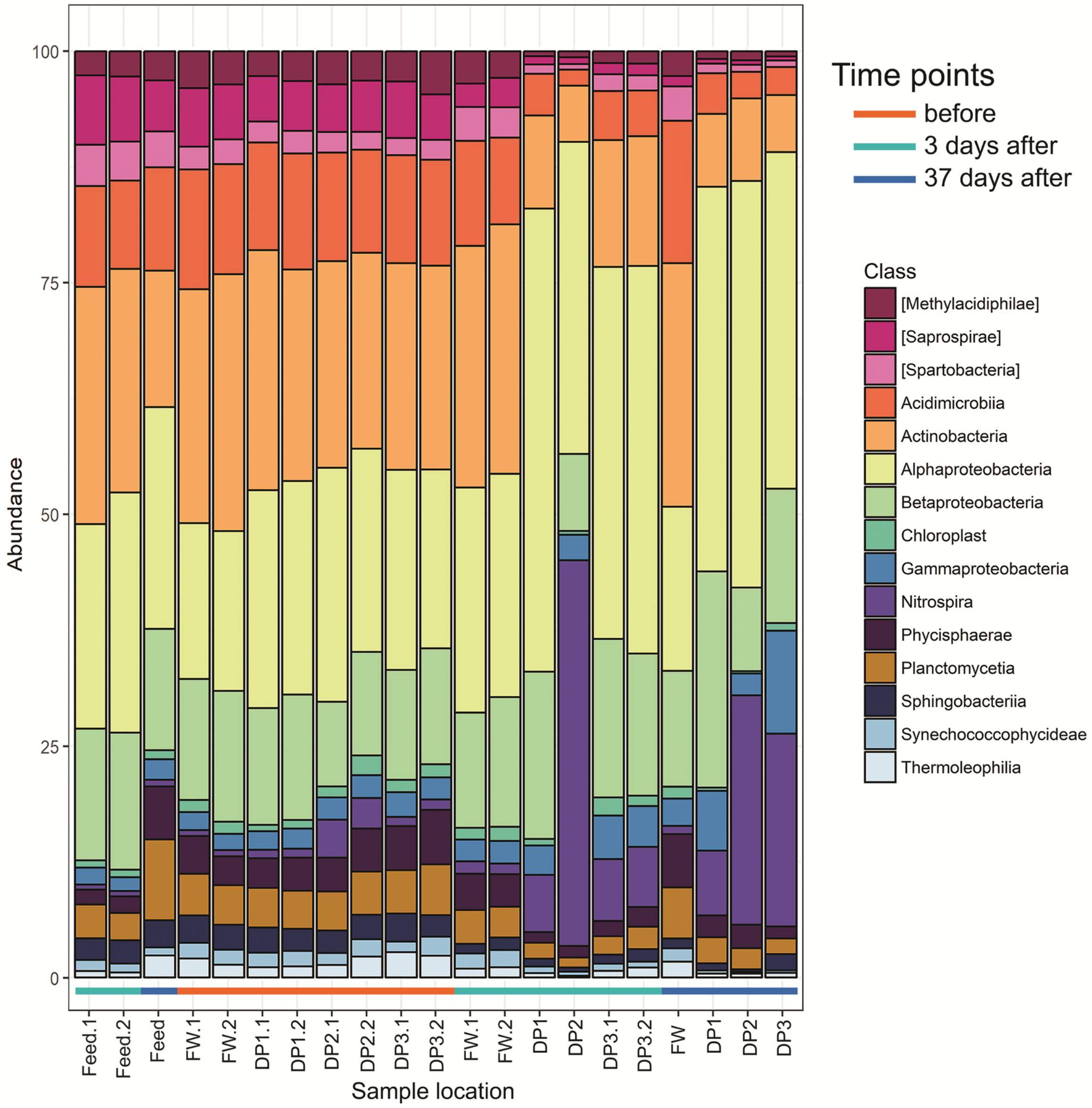

Supplementary Figure 3 – Relative abundance of bacteria in the different samples at the class level. Top 15 most relative abundant classes which contribute to at least 1 % of total reads across all the samples are presented. At each location, one sample was taken before the installation of ultrafiltration (orange line) and two were taken at three and 37 days after the installation (UF1, green line and UF2, blue line). Biological replicates are indicated by a number following the decimal, if included i.e. DP1.1 and DP1.2.

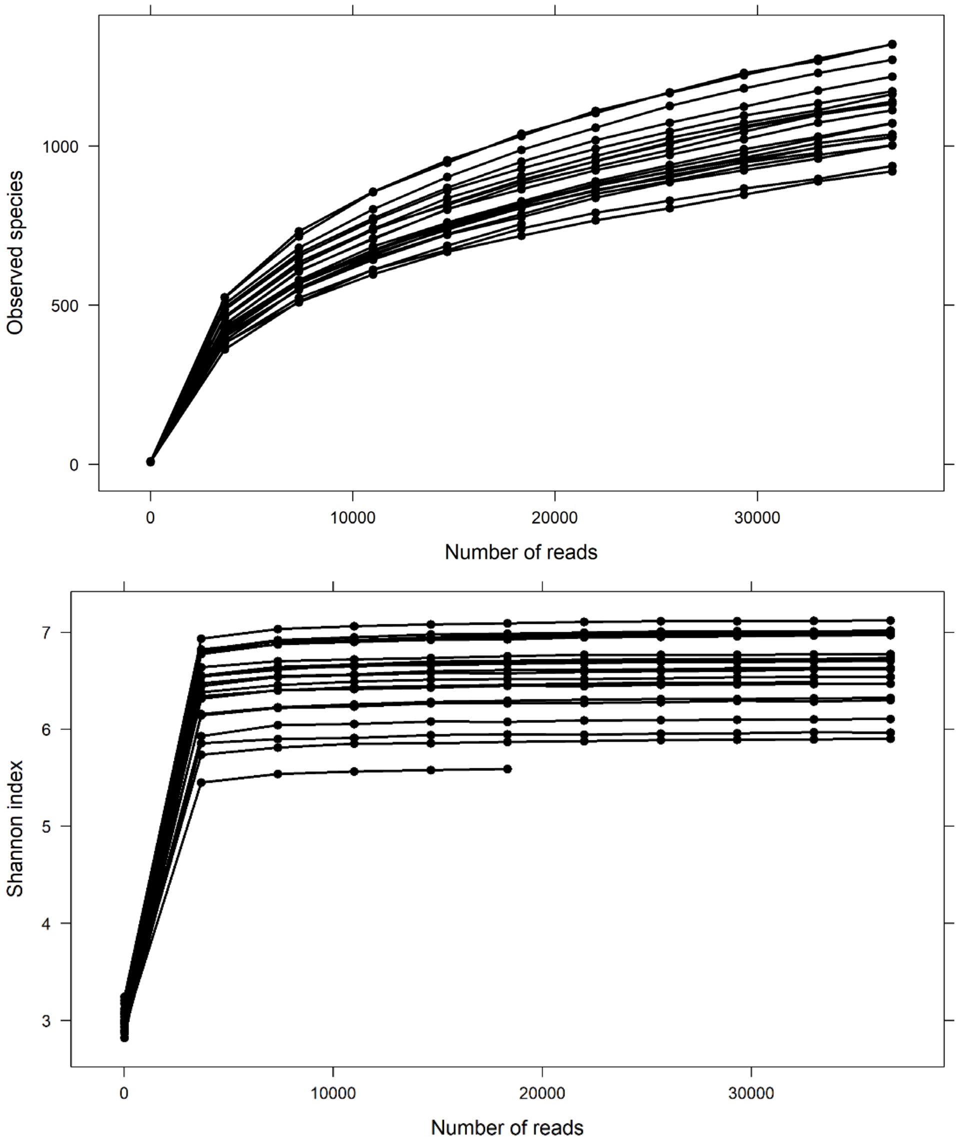

Supplementary Figure 4 – Rarefraction curves based on observed species (number of OTUs) (top) and Shannon index (bottom) for all the 21 samples.

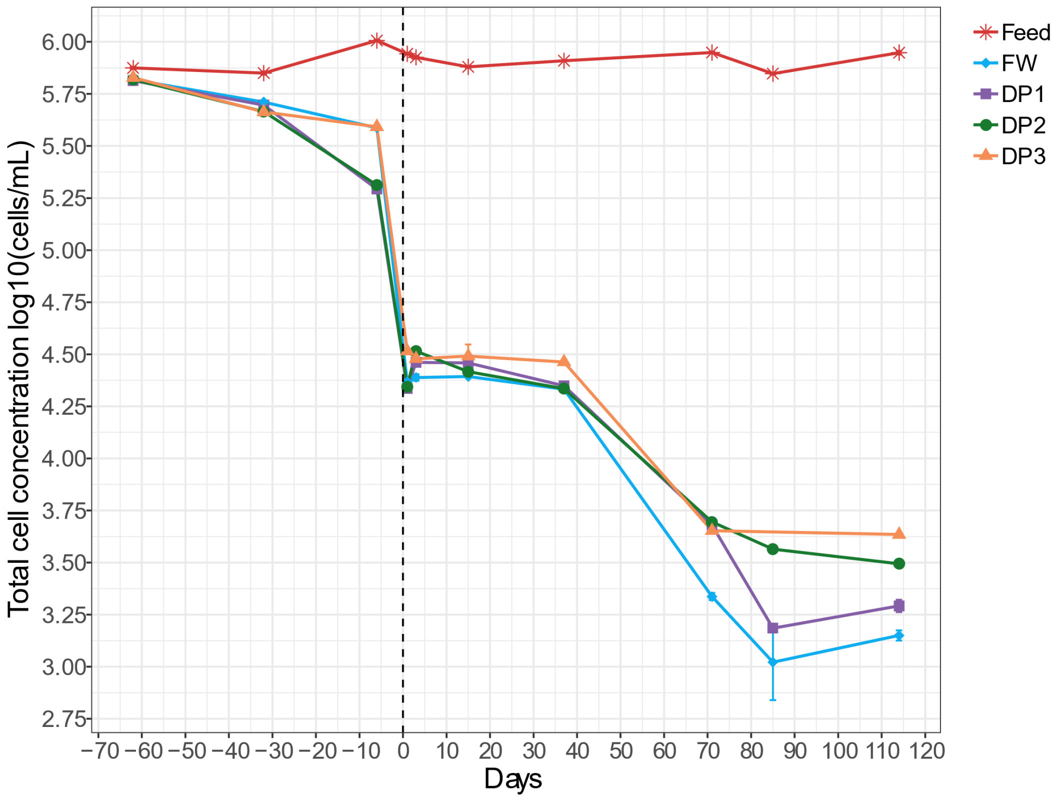

Supplementary Figure 5 – TCC of the distributed water samples before and after UF installation shows an initial drop between days 0 and 37, when feed water was still used for pH regulation, and a final lower plateau, when only UF-treated water was distributed (day 71-114). TCC is shown for feed water to the UF (red line, stars), finished water (FW, blue line, diamonds), and at DP1 (purple line, squares), DP2 (green line, circles) and DP3 (orange line, triangles). Day 0 on the x-axis corresponds to the start of the UF (vertical dashed line). Error bars represent the variation in technical triplicates.

Supplementary Information - Tables

Bacterial release from pipe biofilm in a full-scale drinking water distribution system

Sandy Chan[a,b,c,], Kristjan Pullerits[a,b,c], Alexander Keucken[d,e], Kenneth M. Persson[b,c,d], Catherine J. Paul[a,d*], Peter Rådström[a]

[a]Applied Microbiology, Department of Chemistry, Lund University, P.O. Box 124, SE-221 00 Lund, Sweden

[b]Sweden Water Research AB, Ideon Science Park, Scheelevägen 15, SE-223 70 Lund, Sweden

[c]Sydvatten AB, Hyllie Stationstorg 21, SE-215 32 Malmö, Sweden

[d]Water Resources Engineering, Department of Building and Environmental Technology, Lund University, P.O. Box 118, SE-221 00 Lund, Sweden

[e] Vatten & Miljö i Väst AB, Box 110, 311 22 Falkenberg, Sweden

*****_corresponding author_

<u>Table of Contents</u>

Supplementary Table 1 – Output data from DESeq2 combined with the taxonomic annotation of the different OTUs.

Supplementary Table 2 – Conventional chemical and microbial water quality parameters

Supplementary Table 3 – Natural organic matter content

Supplementary Table 1 – Output data from DESeq2 combined with the taxonomic annotation of the different OTUs.

OTU	baseMean	log2FoldChange	pvalue	padj	Kingdom	Phylum	Class	Order	Family	Genus
**807415**	433.5328	9.4932781	1.43E-09	5.85E-09	Bacteria	Proteobacteria	Alphaproteobacteria	Rhodobacterales	Rhodobacteraceae	Rhodobacter
**New.ReferenceOTU164**	2076.0738	9.3259966	2.71E-06	9.38E-06	Bacteria	Proteobacteria	Alphaproteobacteria	*NA*	*NA*	*NA*
**328951**	1739.4879	8.9535161	2.00E-12	1.13E-11	Bacteria	Nitrospirae	Nitrospira	Nitrospirales	Nitrospiraceae	Nitrospira
**264343**	975.2421	8.8871578	1.15E-12	6.88E-12	Bacteria	Nitrospirae	Nitrospira	Nitrospirales	Nitrospiraceae	Nitrospira
**554981**	984.9402	8.8368927	1.32E-13	9.17E-13	Bacteria	Proteobacteria	Betaproteobacteria	*NA*	*NA*	*NA*
**1143858**	306.662	8.7966979	1.52E-10	7.21E-10	Bacteria	Cyanobacteria	4C0d-2	MLE1-12	*NA*	*NA*
**New.ReferenceOTU371**	230.3143	8.7659308	2.21E-18	2.49E-17	*NA*	*NA*	*NA*	*NA*	*NA*	*NA*
**New.ReferenceOTU202**	341.3616	8.7516688	1.17E-30	2.64E-29	Bacteria	Proteobacteria	Alphaproteobacteria	Rhizobiales	Hyphomicrobiaceae	Hyphomicrobium
**570344**	300.7408	8.5713684	6.28E-16	5.03E-15	Bacteria	Proteobacteria	Alphaproteobacteria	Rickettsiales	*NA*	*NA*
**New.CleanUp.ReferenceOTU32754**	189.374	8.5244266	2.90E-05	9.66E-05	Bacteria	Acidobacteria	Solibacteres	Solibacterales	*NA*	*NA*
**1062748**	598.0293	8.4899176	1.20E-10	6.01E-10	Bacteria	Actinobacteria	Actinobacteria	Actinomycetales	Mycobacteriaceae	Mycobacterium
**4470200**	2036.6014	8.4408388	1.71E-13	1.10E-12	Bacteria	Nitrospirae	Nitrospira	Nitrospirales	Nitrospiraceae	Nitrospira
**233724**	1664.9664	8.2719846	8.54E-21	1.28E-19	Bacteria	Proteobacteria	Alphaproteobacteria	Rickettsiales	*NA*	*NA*
**New.ReferenceOTU24**	404.3908	8.1076291	2.86E-11	1.51E-10	Bacteria	Nitrospirae	Nitrospira	Nitrospirales	Nitrospiraceae	Nitrospira
**New.ReferenceOTU155**	395.014	8.049085	1.68E-08	6.03E-08	Bacteria	Proteobacteria	Gammaproteobacteria	Legionellales	Coxiellaceae	*NA*
**134302**	1951.4365	7.9683312	6.70E-16	5.03E-15	Bacteria	Proteobacteria	Alphaproteobacteria	Rhizobiales	Hyphomicrobiaceae	Hyphomicrobium
**109789**	4244.9859	7.9368478	6.56E-10	2.81E-09	Bacteria	Nitrospirae	Nitrospira	Nitrospirales	Nitrospiraceae	Nitrospira
**568617**	388.9368	7.9347294	1.08E-08	4.05E-08	Bacteria	Proteobacteria	Alphaproteobacteria	Rhodospirillales	Rhodospirillaceae	*NA*
**812978**	274.1116	7.7282295	2.80E-09	1.09E-08	Bacteria	Proteobacteria	Alphaproteobacteria	Rhizobiales	*NA*	*NA*
**New.ReferenceOTU480**	163.8361	7.3352637	1.53E-03	4.76E-03	Bacteria	Proteobacteria	Betaproteobacteria	Burkholderiales	Oxalobacteraceae	*NA*
**564600**	503.6496	7.3197144	1.20E-37	3.59E-36	Bacteria	Proteobacteria	Alphaproteobacteria	Rhizobiales	Hyphomicrobiaceae	Hyphomicrobium
**564309**	646.4925	6.5569246	1.02E-24	1.83E-23	Bacteria	Proteobacteria	Alphaproteobacteria	Rickettsiales	Rickettsiaceae	*NA*
**1108960**	4063.7182	6.1449385	1.56E-67	7.00E-66	Bacteria	Proteobacteria	Alphaproteobacteria	Sphingomonadales	Sphingomonadaceae	Sphingomonas
**1003206**	2011.4925	5.9889776	3.98E-83	3.58E-81	Bacteria	Proteobacteria	Alphaproteobacteria	Sphingomonadales	Sphingomonadaceae	Sphingomonas
**37092**	261.3607	5.5880807	1.76E-10	7.91E-10	Bacteria	Proteobacteria	Alphaproteobacteria	Sphingomonadales	Sphingomonadaceae	Novosphingobium
**692284**	1317.3855	5.5199871	2.84E-18	2.84E-17	Bacteria	Proteobacteria	Betaproteobacteria	Nitrosomonadales	Nitrosomonadaceae	*NA*
**112204**	688.4011	5.3544386	7.60E-18	6.84E-17	Bacteria	Proteobacteria	Betaproteobacteria	Nitrosomonadales	Nitrosomonadaceae	*NA*
**New.ReferenceOTU205**	1093.1424	5.2954801	1.22E-18	1.57E-17	Bacteria	Proteobacteria	Betaproteobacteria	Nitrosomonadales	Nitrosomonadaceae	*NA*

**321513**	232.0724	0.9203116	1.32E-03	4.23E-03	Bacteria	Nitrospirae	Nitrospira	Nitrospirales	Nitrospiraceae	Nitrospira
**New.ReferenceOTU167**	114.5191	-0.471999	1.88E-03	5.63E-03	Bacteria	Proteobacteria	Betaproteobacteria	Burkholderiales	Comamonadaceae	Limnohabitans

Supplementary Table 2 – Conventional chemical and microbial water quality parameters measured before (day -6) and after (day 3 and day 37) the installation of UF. Samples were taken from the different sampling points in the drinking water treatment plant (feed and finished water) and distribution system (DP1, DP2 and DP3).

**Sampling day**	**-6**					**3**					**37**				
**Sampling point**	**Feed**	**Finished water**	**DP1**	**DP2**	**DP3**	**Feed**	**Finished water**	**DP1**	**DP2**	**DP3**	**Feed**	**Finished water**	**DP1**	**DP2**	**DP3**
**Alkalinity (mg HCO_3/L)**	22	56	55	55	54	19	58	61	62	61	20	61	61	61	61
**Aluminium (mg/L)**	0.014	0.014	0.017	0.016	0.018	0.016	0.029	0.029	0.031	0.029	0.017	0.024	0.023	0.024	0.025
**Ammonium (mg/L)**	0.005	0.062	0.035	0.033	0.026	0.005	0.049	0.027	0.023	0.03	0.005	0.058	0.027	0.042	0.022
**Ammonium-nitrogen (mg/L)**	0.005	0.048	0.027	0.026	0.02	0.005	0.038	0.021	0.018	0.023	0.005	0.045	0.021	0.033	0.017
**COD-Mn (mg O_2/L)**	2.3	2.1	2.1	2.1	2.1	1.9	1.2	1.2	1.3	1.2	2.3	1.3	1.3	1.3	1.4
**DOC (mg/L)**	2.8	2.5	2.4	2.5	2.7	2.8	1	1	1	1	2.5	1	1	1	1
***Escherichia coli* (CFU/100 mL)**	0.5	0.5	0.5	0.5	0.5	0.5	0.5	0.5	0.5	0.5	0.5	0.5	0.5	0.5	0.5
**Phosphorus (mg/L)**	0.0025	0.0025	0.0062	0.0058	0.0025	0.0025	0.0025	0.0025	0.0077	0.0025	0.0025	0.0025	0.0025	0.0025	0.0025
**Color 410 nm (mg Pt/L)**	13	11	9.5	8.7	12	11	2.5	2.5	2.5	2.5	11	2.5	2.5	2.5	2.5
**Iron (mg/L)**	0.01	0.01	0.01	0.01	0.01	0.029	0.01	0.01	0.01	0.01	0.021	0.01	0.01	0.01	0.01
**Calcium (mg/L)**	8.2	19	20	20	19	7.5	21	21	21	21	7.6	21	22	21	22
**Coliforms (CFU/100 mL)**	0.5	0.5	0.5	0.5	0.5	0.5	0.5	0.5	0.5	0.5	0.5	0.5	0.5	0.5	0.5
**Conductivity (mS/m)**	11	16	16	16	16	11	17	17	17	17	11	17	17	17	17
**Copper (mg/L)**	0.01	0.01	0.01	0.01	0.01	0.01	0.01	0.01	0.026	0.01	0.01	0.01	0.01	0.026	0.01
**Heterotrophic bacteria 7 days (CFU/mL)**	17	0.5	0.5	5	6	29	0.5	1	3	22	18	0.5	0.5	3	3
**Magnesium (mg/L)**	2.3	2.4	2.5	2.4	2.4	2.2	2.2	2.2	2.2	2.1	2.4	2.4	2.4	2.3	2.3
**Manganese (mg/L)**	0.06	0.059	0.056	0.053	0.05	0.034	0.033	0.033	0.033	0.029	0.014	0.013	0.013	0.012	0.012
**Nitrate (mg/L)**	1.5				1.5	1.5	1.9	1.9	1.4	1.3	1.5	1.4	1.4	1.5	1.4
**Nitrite (mg/L)**	0.035	0.0035	0.069	0.046	0.079	0.035	0.0035	0.059	0.03	0.056	0.035	0.0035	0.069	0.036	0.072
**Heterotrophic bacteria 3 days (CFU/mL)**	13	0.5	0.5	0.5	2	10	0.5	0.5	0.5	0.5	8	0.5	0.5	0.5	0.5
**pH**	7.5	8	8.2	8.1	8	7.3	8	8.1	8	8	7.3	8.1	8.2	8.1	8
**TOC (mg/L)**	2.9	2.7	2.7	2.7	2.7	2.8	2	2.1	2.1	2	2.7	1	1	1	1
**Hardness (°dH)**	1.7	3.1	3.3	3.3	3.2	1.6	3.4	3.5	3.4	3.4	1.6	3.5	3.6	3.5	3.6
**Turbidity (FNU)**	0.21	0.05	0.29	0.3	0.2	0.22	0.14	0.48	0.19	0.19	0.23	0.05	0.05	0.05	0.11
**Temperture (°C)**	6.9	6.7	7.4	9	8.2	6.2	6.3	7.2	8.7	7.3	5.7	5.7	6.8	8.2	6.7

Supplementary Table 3 – Natural organic matter content before (day -6) and after (day 3 and day 37) the installation of UF for the different sampling points in the drinking water treatment plant (feed and finished water) and distribution system (DP1, DP2 and DP3).

**Sampling day**	**-6**					**3**					**37**				
**Sampling point**	**Feed**	**Finished water**	**DP1**	**DP2**	**DP3**	**Feed**	**Finished water**	**DP1**	**DP2**	**DP3**	**Feed**	**Finished water**	**DP1**	**DP2**	**DP3**
**DOC (Dissolved OC) (ppb-C)**	2442	2308	2312	2321	2360	2445	1817	1871	1826	1821	2391	1674	1600	1608	1611
**HOC (Hydrophobic OC) (ppb-C)**	n.q.	n.q.	71	n.q.	38	n.q.	126	101	88	25	n.q.	81	n.q.	14	14
**Inorganic colloids (m^{-1})**	n.q.	n.q.	n.q.	n.q.	n.q.	n.q.	n.q.	n.q.	n.q.	n.q.	n.q.	n.q.	n.q.	n.q.	0.03
**SUVA (L/mg*m)**	3.15	2.99	3.1	3.24	3.08	3.29	2.39	2.4	2.55	2.66	3.34	2.44	2.45	2.34	2.52
**CDOC (Chromatographic DOC) (ppb-C)**	2442	2308	2241	2321	2322	2445	1690	1770	1738	1796	2391	1593	1600	1595	1598
Biopolymers (ppb-C)	65	45	27	38	39	65	24	34	30	30	57	31	35	29	25
Dissolved organic nitrogen (DON) (ppb-N)	7	5	4	5	4	6	n.q.	n.q.	4	5	6	4	4	n.q.	n.q.
N/C ratio (µg/µg)	0.1	0.11	0.14	0.14	0.11	0.09	-	-	0.12	0.15	0.11	0.12	0.1	-	-
% proteins (% of biopolymer)	31	33	41	41	33	27	-	-	36	45	34	35	31	-	-
Humic substances (ppb-C)	1487	1472	1421	1466	1475	1564	957	970	956	983	1562	935	946	942	954
Dissolved organic nitrogen (DON) (ppb-N)	31	32	32	32	32	31	21	22	23	22	32	20	21	19	20
N/C ratio (µg/µg)	0.02	0.02	0.02	0.02	0.02	0.02	0.02	0.02	0.02	0.02	0.02	0.02	0.02	0.02	0.02
Aromaticity (L/mg*m)	3.71	3.5	3.64	3.56	3.56	3.89	3.02	2.99	3.04	2.87	4.11	2.39	2.4	2.42	2.43
Molecular weight (g/mol)	647	566	640	581	601	606	523	525	522	524	685	476	477	482	478
Building blocks (ppb-C)	509	422	478	445	456	448	425	442	433	442	431	340	336	332	340
LMW Neutrals (ppb-C)	509	355	292	352	332	350	276	312	304	330	309	288	283	292	278
LMW Acids (ppb-C)	24	13	24	20	19	18	8	12	15	11	32	0	n.q.	n.q.	0

n.q. = not quantifiable (< 1 ppb;signal to noise ratio)
